# Exploring the effects of fitbit incentive on treatment outcomes in veterans undergoing intensive pain rehabilitation program

**DOI:** 10.1186/s41687-024-00721-z

**Published:** 2024-04-09

**Authors:** Tiffany Toor, Sarah Palyo, Kathryn Schopmeyer, Alan N. Simmons, Irina A. Strigo

**Affiliations:** 1 Department of Veterans Affairs (VA) San Francisco Healthcare System, San Francisco, CA USA; 2 University of California, San Francisco, CA USA; 3 Department of Veterans Affairs (VA), San Diego Healthcare System, San Diego, CA USA; 4 Emotion and Pain Laboratory, San Francisco Veterans Affairs Health Care Center, 4150 Clement Street, 94121 San Francisco, CA USA

**Keywords:** Chronic pain, Fitbit, Wearable electronic devices, Treatment satisfaction, Patient satisfaction, Pain treatment, Veteran

## Abstract

**Objective:**

This study compares clinical pain outcomes between patients in a pain treatment program that received a Fitbit, to patients that did not. We also explored: (1) cognitive, emotional, and psychological factors that may have impacted the decision to opt in to receiving a Fitbit; and (2) whether the choice to receive a Fitbit impacted changes in cognitive, emotional, and psychological factors following treatment.

**Methods:**

Among 58 patients in a multidisciplinary pain treatment program at a Veterans Affairs Healthcare System hospital, 31 patients opted to receive a Fitbit as adjunct treatment, while 27 did not. This study utilized patient-reported and practitioner-collected data from the pain treatment program.

**Results:**

Compared to the non-Fitbit group, the Fitbit group displayed a significant decrease in average pain intensity, however showed no correlation between Fitbit activity and average pain intensity. Additionally, treatment satisfaction was the only predictor of treatment group, when modeling pre- and post-treatment outcomes changes.

**Conclusion:**

The implementation of a Fitbit may lead to improved pain intensity. Initial evidence suggests that opting to receive a Fitbit during a pain treatment program indicates treatment engagement leading to greater treatment satisfaction. Future work is needed to verify and expand upon this potential mechanism.

**Supplementary Information:**

The online version contains supplementary material available at 10.1186/s41687-024-00721-z.

## Introduction

Chronic pain is a highly prevalent condition and is among the most common reasons why adults seek medical care [1]. Pain is understood to be a multidimensional experience, affected by biological changes, genetic vulnerabilities, psychological factors, and socioeconomic components [2]. Thus, the biopsychosocial model, in which a team-based variety of therapeutic modalities are used for treating pain has been widely accepted as the main heuristic [2, 3]. The Department of Veterans Affairs has seen as rise in interdisciplinary pain treatment programs owing largely to the 2009 Veterans Health Administration (VHA) Directive [4], which established a new standard of multimodal pain care founded on a biopsychosocial patient-centered approach. An Intensive Pain Rehabilitation Program (IPRP) consists of several treatment components including individual and team meetings with providers, attending group classes on Acceptance and Commitment Therapy, Cognitive-Behavioral Therapy, physical therapy, pharmacological and nutritional counseling, and pain education [5, 6]. The effectiveness of IPRP is evidenced by the reductions in pain-related domains of functioning, pain catastrophizing, and in sleep-related difficulties [5, 6]. One goal of IPRP, within the modality of physical therapy, is to address physical activity that may improve clinical pain outcomes such as functioning, sleep, and fearful thoughts related to pain.

Wearable monitoring devices, such as Fitbits, have shown to be a useful and practical method of monitoring physical activity in populations with chronic illnesses [7–10]. For patients with chronic pain, wearable monitoring devices have been found to increase treatment adherence [11, 12], although interpretation is obscured by dropouts and substantial missing data on physical activity [13, 14]. While adherence has proven to vary across studies, several studies have found that pain management programs using wearable monitoring devices fail to find significant increases in physical activity, such as step-count, in the groups receiving wearable monitoring devices, or significant differences in physical activity compared to control groups (i.e., standard pain management care with information provided on physical activity, but no monitoring device provided [13]). Increased physical activity is associated with reduced pain intensity in chronic pain patients [15, 16], and in patients with low pain catastrophizing [17]. Conversely, increased physical activity is associated with increased pain intensity, in those with high pain [17]. The benefit of wearable monitoring devices can be maximized if paired with skills training to overcoming barriers to engagement in physical activity [18].

To evaluate how wearable monitoring devices may be integrated with IPRP to improve treatment outcomes, the San Francisco Veterans Affairs Health Care System (SFVAHCS) disseminated Fitbits to patients, provided instruction on the use of the device, implemented Fitbit goal setting by patients (such as daily step goals), and tracked progress using weekly logs to be filled out by patients regarding activity tracking. The implementation of the Fitbit in SFVAHCS IPRP was an available add-on for participants who expressed interest and did not change any of the existing IPRP components [6].

The primary goal of the present observation study was to compare clinical pain outcomes between patients in a pain treatment program that received a Fitbit, compared to patients in the same pain treatment program that did not receive a Fitbit. We also explored whether: (1) cognitive, emotional, and psychological factors that may have impacted people’s decision to opt into receiving a Fitbit during the IPRP treatment; and (2) the choice to receive Fitbit impacted changes in cognitive, emotional, and psychological factors following treatment. An evolving understanding of the relationships between these factors in a veteran sample undergoing such integrated rehabilitation may further inform treatment development, optimization, assessments, and protocols.

## Methods

**Fig. 1:**
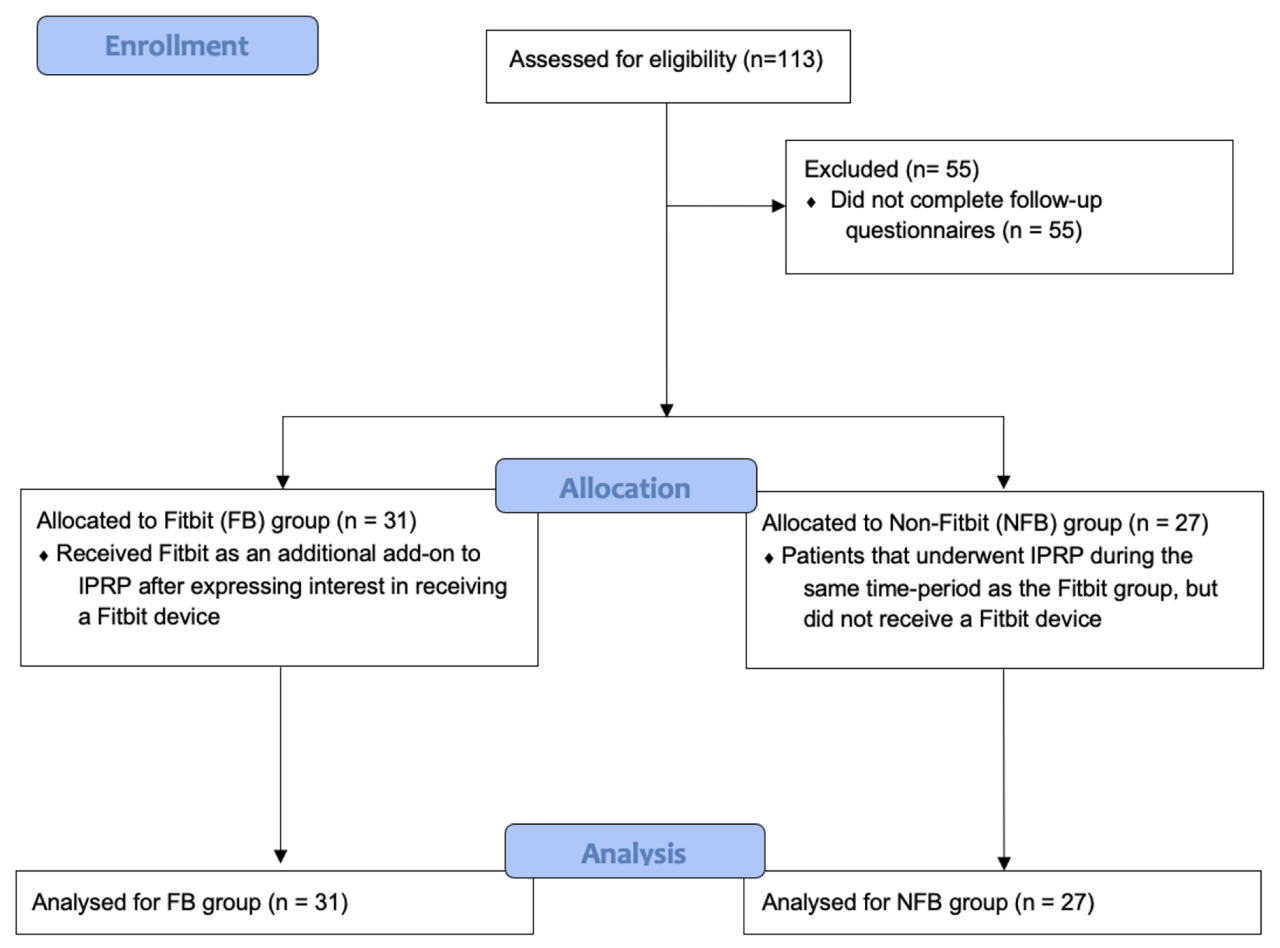
Consort diagram of study flow

Study procedures were approved by the SFVAHCS and University of California San Francisco Institutional Review Boards. Patient data was gathered retrospectively from Veterans screened for enrollment into the Intensive Pain Rehabilitation Program (IPRP) between 2015 and 2018. The SFVAHCS IPRP program is an intensive and interdisciplinary treatment program designed for patients receiving care through the SFVAHCS who suffer from functionally impairing chronic, non-cancer pain conditions. Inclusion in the program requires a referral from clinician within the Veterans Affairs Healthcare System and an IPRP team-based evaluation to determine an individual’s fit for the program. All potential participants are referred to the IPRP by their primary care provider, or other relevant specialty care provider (e.g., neurologist, neurosurgeon, rheumatologist, podiatrist, orthopedic surgeon). All referrals complete the screening package (see below). Participants are all VA-enrolled and include Veterans as well as non-Veteran beneficiaries who are eligible for VA care (e.g., spouses of 100% service-connected Veterans). Of the 113 patients who were screened between July 2015 and March 2018, patients were given the option to receive a wearable Fitbit device as additional adjunct treatment to IPRP. Only those patients who expressed an interest in receiving the Fitbit device were given one. Of the patients that underwent IPRP during this time frame, individuals were excluded from this data analysis if: (1) they dropped out, (2) they completed the program but had not completed post-treatment questionnaires, (3) more than 15% of a questionnaire was missing either pre- or post- treatment, (4) they were not Veterans, or (5) they were re-enrolled in the program (only the first enrollment was included in analyses to control for repetition effects). The final sample included 58 subjects. Please see the CONSORT diagram (Fig. 1) for the study flow.

### Measures

#### Self-report questionnaires

Patients self-reported clinical and demographic information both pre- and post- treatment. Information included age, sex, race, years of education completed, duration of pain, and identified pain sites. Additionally, average, or “usual” pain intensity (during the past week) and “acceptable” pain level was reported using a numeric pain rating scale (NPRS) (0–10). Of note, “acceptable” level of pain refers to an anticipated pain score with which a patient would be comfortable rather than acceptance of their pain condition. Here, it is a measure of patients’ expectations regarding treatment. Outpatient doses of opioids were standardized to morphine equivalent daily dose (MEDD), calculated according to conversion factors obtained from the CDC [19]. Finally, patients completed a brief questionnaire on treatment satisfaction, from which a total score ranging from 0 to 50 was calculated. Due to a lack of normal distribution (skew ∼ 1.56), we transformed the treatment satisfaction score into a binary 0 or 1, based on whether they indicated 0 satisfaction or some satisfaction.

##### The pain outcomes questionnaire-for veterans (PDQ-VA) [20]

is a multidomain instrument to assess pain, specifically developed and validated for veterans. The POQ-VA scale includes the major pain-related domains of functioning, including pain intensity via the 0–10 pain numeric rating scale (pain NRS), pain interference in activities of daily living (ADL), interference in mobility (MOB), negative affect (NA), vitality (VIT), and pain-related fear (Fear). The POQ-VA scale has been shown to have high internal reliability, strong generalizability, and good discriminant and concurrent validity, as well as demonstrated sensitivity to treatment-related change [20].

##### The pain catastrophizing scale (PCS) [21]

is a 13-item self-report measure used to assess an individual’s negative cognitions accompanying the experience or anticipation of pain. It is composed of three subscales, representing rumination (e.g., “I can’t seem to keep it out of my mind”), magnification (e.g., “I wonder whether something serious may happen”), and helplessness (e.g., “There’s nothing I can do to reduce the intensity of the pain”). Each item is rated using a 5-point Likert scale (0 representing “not at all” and 4 representing “all the time”). The total score depicts a single representation of general pain catastrophizing, with higher scores indicating a greater degree of pain-related catastrophic thinking. The PCS is a widely used among various chronic pain populations and has shown good reliability and validity [22].

##### The insomnia severity index (ISI) [23]

is a 7-item self-report measure used to measure the subject’s perceived severity of “current” (i.e., last 2 weeks) insomnia symptoms, such as falling asleep, staying asleep, and waking early. Each item of the ISI is scored on a 5-point Likert scale ranging from 0 (“no problem”) to 4 (“very severe problem”), yielding a total score ranging from 0 to 28 (i.e., no clinically significant insomnia to severe insomnia). The ISI has been shown to have adequate psychometric properties (i.e., internal consistency, concurrent validity, factor structure) and can be used as a reliable and valid instrument to evaluate insomnia [24].

#### Physical therapy measures

Objective physical function was assessed using four physical therapy (PT) measures: (1) the 5 time sit-to-stand (5TSTS) test, (2) the 6 m Timed Up and Go (TUG) test, (3) the multi-directional reach (MDR) test in the front, back, right, and left directions, and (4) the 6-minute walk (test). For the purposes of data analysis, the MDR test was averaged between the four directions into one value. All four physical function tests have been validated for use with adults, including older adults, and those with chronic pain [25, 26]. PT tests were conducted at the start and end of the IPRP treatment.

#### Fitbit measures

The wearable Fitbit devices collected the following data: steps taken, distance travelled, floors climbed, and calories burnt. For the purposes of this study and to use the most completed data, we chose to analyze the Fitbit data using steps taken. In order to understand how engaged the participants were with using the Fitbit devices, we calculated their “level of engagement” using the average days per week that each participant wore the Fitbit. If there was any Fitbit usage on a particular day, that day was counted in the calculation for the average number of days the Fitbit was worn per week.

#### Data Analysis

All statistical analyses were performed using the statistical program R version 4.0.2, RStudio, & JASP version 0.14.1. To understand the effects of receiving a Fitbit device in a pain treatment program, patients were categorized into 2 groups -- ‘Fitbit’ (FB) and ‘Non-Fitbit’ (NFB)– based on whether they were interested in receiving a Fitbit device. Up to 20 Fitbits were available, so any enrolled patient that wanted a Fitbit received one, up to 20 subjects enrolled concurrently (maximum was never utilized). To compare the questionnaire data, the comparison group consisted of a similar number of patients who participated in the same interdisciplinary pain treatment program during the same timeframe but did not elect to receive a Fitbit device. Demographic and questionnaire data were compared between groups using parametric T-tests and nonparametric Mann-Whitney U tests, depending on whether the variable was normally distributed or not, respectively. Between-group analyses were conducted using repeated measures ANOVA tests. Correlation tests were used in the Fitbit groups to determine the relationship between average steps and psychological measures. We used logistic regression, with both the baseline measures and the post-treatment outcomes, to model associations between treatment group and various demographic, clinical, and psychological factors. Unadjusted odds ratios from these models are presented in the results. The data that support the findings of this study are available upon request from the authors.

## Results

In order to explore the potential benefits of the addition of a Fitbit to IPRP, patients were categorized into two groups– Fitbit (FB) and Non-Fitbit (NFB). All participants were offered Fitbit after the enrollment in the program and prior to completing baseline measures package. Therapists in the program were blind to the group assignment. Thirty-one individuals opted to receive a Fitbit and were classified as the FB group, while 27 did not and thus were classified into NFB group consisting of 27 individuals.

### Demographic and baseline characteristics

The FB and NFB groups did not significantly differ in age, sex, race, marital status, years of education, or employment status (*p*’s > 0.05; see Table 1). Despite no statistically significant difference, slight differences existed. Specifically, the FB group was 64.5% male and 35.5% female, while the NFB group was 81.5% male and 18.5% female. In terms of race, the FB group was 71.0% White while the NFB group was 44.4% White. Additionally, the mean age of the FB group was slightly younger (*M* = 51.1) than the NFB group (*M* = 57.6).

The two groups were also compared in terms of their baseline average pain, as well their self-reported measure of acceptable pain. The two groups did not significantly differ in baseline average pain (FB *M* = 5.9; NFB *M* = 6.4; *p* = 0.387), nor did the groups differ significantly in their ratings of acceptable pain (FB *M* = 3.2; NFB *M* = 3.6; *p* = 0.401). Additionally, the groups did not differ significantly in any of the collected physical therapy measures (*p*’s > 0.05; see Table 2).

**Table 1:** Veteran Sample Demographic Characteristics (*n* = 58)

	Fitbit) (*N* = 31)	No Fitbit (*N* = 27)	*p*
Age– mean (σ)	51.1 (14.4) ^#^	57.6 (11.6)	*p* = 0.070
Sex– no. of participants (%)			*p* = 0.226
*Male*	20 (64.5)	22 (81.5)	
Race– no. of participants (%)			*p* = 0.438
*African American*	4 (12.9)	6 (22.2)	
*White*	22 (71.0)	12 (44.4)	
*Hispanic*	2 (6.5)	3 (11.1)	
*Asian*	0 (0.0)	1 (3.7)	
*Other*	2 (6.5)	3 (11.1)	
*Multiple Races*	1 (3.2)	2 (7.4)	
Marital status– no. of participants (%)			*p* = 0.250
*Never married*	5 (16.1)	11 (40.7)	
*Married*	17 (54.8)	11 (40.7)	
*Living with someone but not married*	2 (6.5)	0 (0.0)	
*Divorced or separated*	4 (12.9)	4 (14.8)	
*Widowed*	2 (6.5)	1 (3.7)	
*Other*	1 (3.2)	0 (0.0)	
Employment status– no. of participants (%)			*p* = 0.393
*Full-time*	2 (6.5)	2 (7.4)	
*Part-time*	2 (6.5)	2 (7.4)	
*Unemployed, not interested in returning to work*	1 (3.2)	1 (3.7)	
*Unemployed, looking for work*	1 (3.2)	1 (3.7)	
*Unemployed, disabled*	14 (45.2)	7 (25.9)	
*Retired, due to pain*	4 (12.9)	9 (33.3)	
*Retired, not due to pain*	2 (6.5)	4 (14.8)	
*Other*	5 (16.1)	1 (3.7)	
Years of Education– mean (σ)	14.1 (2.4)	14.1 (2.3)	*p* = 0.909
Baseline Average Pain - mean (σ)	5.9 (2.1)	6.4 (2.0)	*p* = 0.387
Acceptable Pain - mean (σ)	3.2 (2.1)	3.6 (1.7)	*p* = 0.401
Baseline Morphine Equivalent Daily Dose (MEDD) - mean (σ)	28.1 (64.7)	22.5 (42.9)	*p* = 0.638^*^

Legend:

^*^: Mann-Whitney U-test, used as a non-parametric test for data not normally distributed. All other p-values calculated using Independent Samples T-Test for normally distributed data

^#^: *n* = 30. Baseline average pain and acceptable pain reported by patients on a scale of 0–10. Treatment satisfaction reported by patients on a total scale of 0–50

Bold indicates results significantly different (*p* < 0.05) between the two craving groups. Percentages may not add to 100% due to rounding. Sub-categories marked as “other” refer to patients that responded with more than one option to a demographic question

**Table 2:** Physical Therapy Measures (*n* = 58)

		Fitbit (*N* = 31)	No Fitbit (*N* = 27)	*p*
**Pre-Treatment**	Timed Up and Go (seconds) - mean (σ)	10.7 (3.2)	12.2 (6.9)	*p* = 0.262
	5x Sit-to-Stand (seconds) - mean (σ)	24.8 (15.4)	25.4 (20.6)	*p* = 0.906
	MDR-F (inches) - mean (σ)	9.3 (3.6)	9.7 (3.8)	*p* = 0.690
	MDR-B (inches) - mean (σ)	6.2 (2.1)	5.4 (2.2)	*p* = 0.157
	MDR-R (inches) - mean (σ)	5.7 (2.1)	6.1 (2.4)	*p* = 0.522
	MDR-L (inches) - mean (σ)	5.9 (2.1)	6.1 (2.1)	*p* = 0.774
	6 min Walk (feet) - mean (σ)	1386.2 (556.8)^#^	1762.2 (1140.1)^^^	*p* = 0.166
**Post-Treatment**	Timed Up and Go (seconds) - mean (σ)	8.0 (2.4)	8.8 (2.5)	*p* = 0.217
	5x Sit-to-Stand (seconds) - mean (σ)	13.6 (5.8)	14.7 (4.2)	*p* = 0.432
	MDR-F (inches) - mean (σ)	11.4 (2.7)	11.3 (3.3)	*p* = 0.878
	MDR-B (inches) - mean (σ)	8.2 (2.8)	7.0 (2.1)	*p* = 0.069
	MDR-R (inches) - mean (σ)	8.7 (3.0)	8.2 (2.5)	*p* = 0.479
	MDR-L (inches) - mean (σ)	7.9 (2.7)	7.9) (2.3)	*p* = 0.951
	6 min Walk (feet) - mean (σ)	1680.9 (327.4)^#^	1719.8 (793.2)^^^	*p* = 0.828

Legend:

^#^: *n* = 25

^^^: *n* = 16

All *p*-values calculated using Independent Samples T-Test for normally distributed data

### Primary outcome: average reported pain

**Fig. 2:**
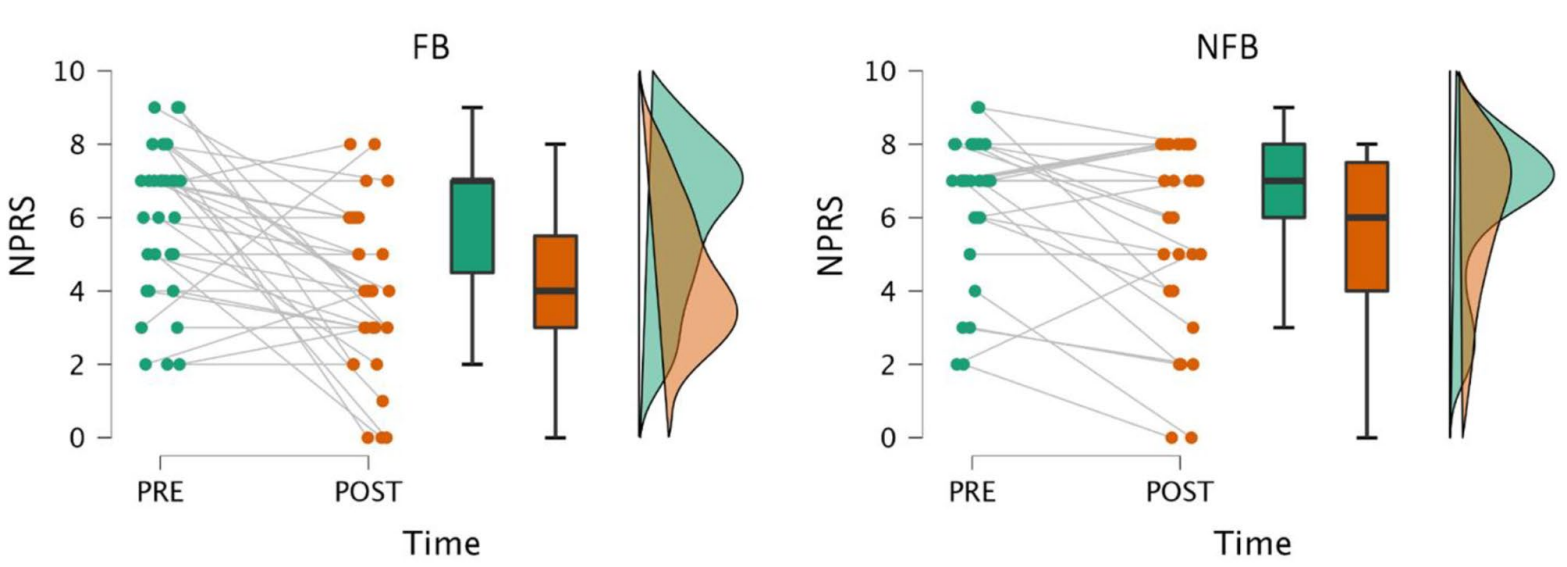
Raincloud plots of average pain, pre- and post-treatment, across groups. A significant difference was found between groups when comparing change in average pain across time– pre- vs. post-treatment. Average pain is displayed using the Numeric Pain Rating Scale (NPRS). The raincloud plots display the raw data (colored dots), mean values and 95% confidence intervals (black lines on the box plots), and probability distributions (vertical “clouds”). The plot on the left depicts changes in the FB group (*N* = 31), while the plot on the right depicts changes in the NFB group (*N* = 27)

The primary goal was to examine whether changes in clinical pain outcomes differed between the two treatment groups– FB and NFB. As determined by the repeated measures ANOVA with group (FB, NFB) as a between-subject factor and time (pre, post) as a within-subject repeated measure, we found a significant group (F(1,56) = 4.452, *p* = 0.039) and time (F(1,56) = 20.143, *p* = 0.000) but not group by time (F(1,56) = 2.567, *p* = 0.115) effects on the reported average pain intensity. Holm-Sidak post-hoc analysis revealed that the FB group showed a greater decrease in average pain intensity compared to the NFB group, with a mean difference of 1.007 on the 0–10 NPRS. This comparison is plotted in Fig. 2. Additionally, the mean change in average pain, from start of treatment to the end, was larger in the FB group (-2.032) compared to the NFB group (-0.963), but statistically this was not significant (*p* = 0.128). Nevertheless, within the FB group neither the average daily steps (Spearman’s ρ = 0.217, *p* = 0.241) nor their level of engagement (average number of days Fitbit worn) (Spearman’s ρ = 0.301, *p* = 0.100) correlated significantly with the changes in pain scores over time.

### Exploratory regression analyses

**Fig. 3:**
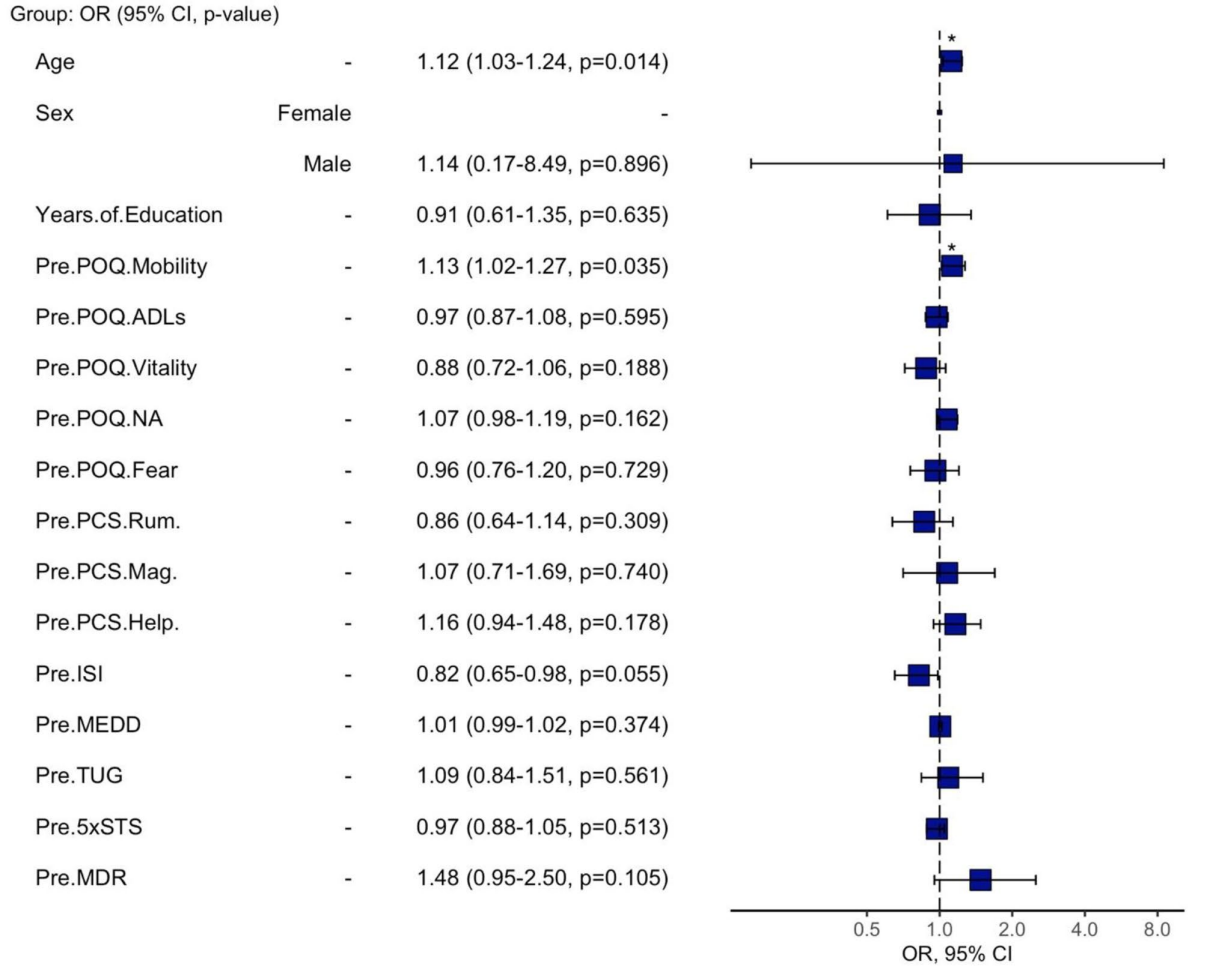
Odds ratio plot of pre-treatment measures. Odds ratio plot of the explanatory baseline variables included in the exploratory logistic regression analysis for predicting treatment group. The blue squares represent actual odds ratios, and the bars denote 95% confidence intervals

**Fig. 4:**
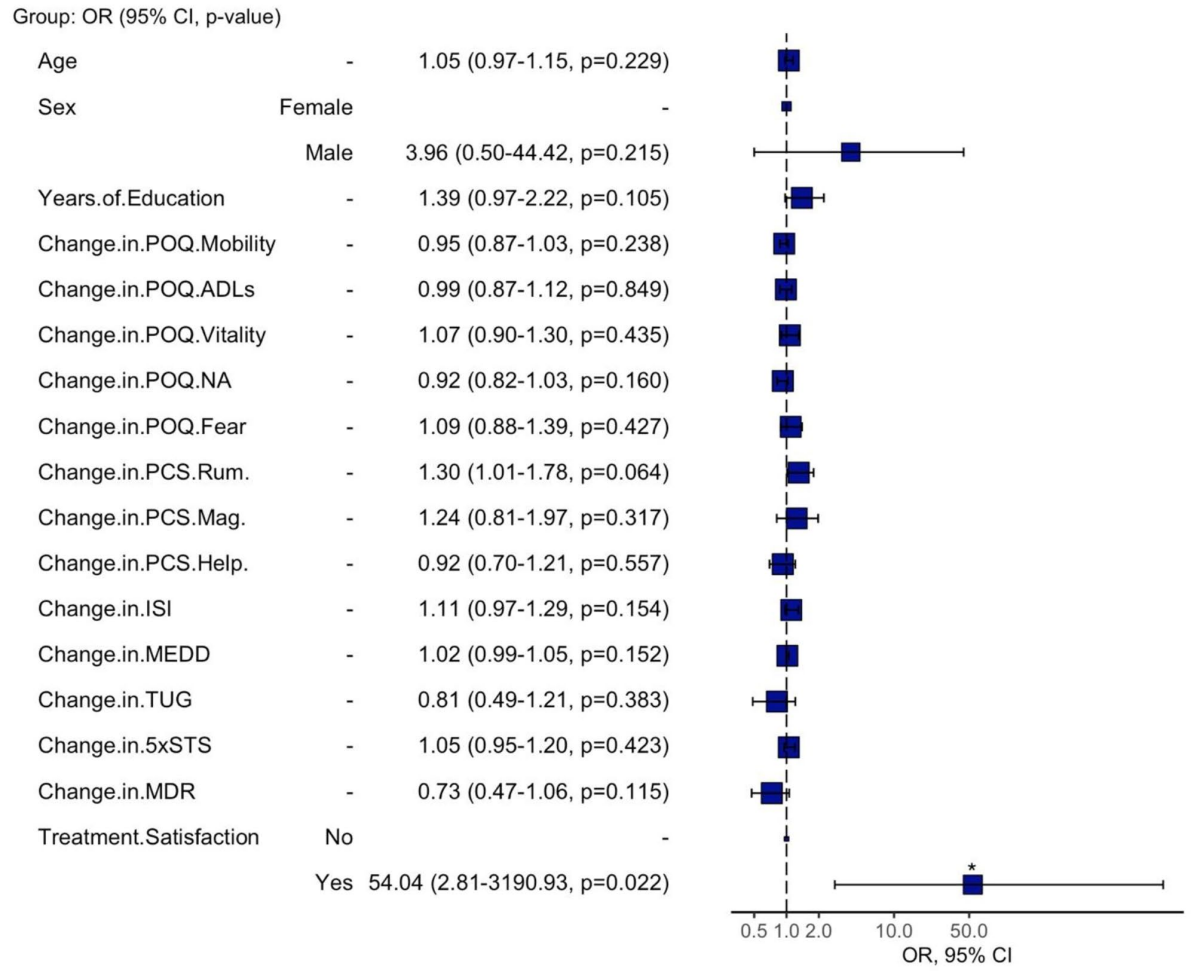
Odds ratio plot of changes between pre- and post-treatment. Odds ratio plot of the explanatory change-in-treatment variables included in the exploratory logistic regression analysis for predicting treatment group. The blue squares represent actual odds ratios, and the bars denote 95% confidence intervals

We explored whether the choice to receive a Fitbit was related to baseline and/or impacted changes in cognitive, emotional, and psychological factors following treatment. The results of exploratory logistic regression analysis are reported using odds ratios in Fig. 3 for pre-treatment measures & Fig. 4 for changes between pre- and post-treatment outcomes. When using the pre-treatment measures, age (odds ratio = 1.12, *p* = 0.014) and POQ mobility (odds ratio = 1.13, *p* = 0.035) scores significantly predicted treatment group, but the model overall was not statistically significant (*p* > 0.05). The second model, which examined changes between pre- and post-treatment outcomes was statistically significant (*p* = 0.040) and demonstrated that treatment satisfaction scores (odds ratio = 54.04, *p* = 0.022) were a significant predictor of treatment group.

## Discussion

The primary goal of this study was to compare clinical pain outcomes between patients in a pain treatment program that received a Fitbit, compared to patients in the same pain treatment program that did not receive a Fitbit. We also explored whether: (1) cognitive, emotional, and psychological factors that may have impacted people’s decision to opt into receiving a Fitbit during the IPRP treatment; and (2) the choice to receive Fitbit impacted changes in cognitive, emotional, and psychological factors following treatment.

The two study groups– FB and NFB– did not differ significantly in any baseline characteristics, including demographic variables and physical therapy measures. These demographic variables included sex, race, marital status, years of education, or employment status. Despite no statistically significant difference in these demographics, it is noteworthy to mention that compared to the NFB group, the FB group had proportionally more females, more White subjects, and was slightly younger in age. Additionally, despite no statistically significant difference in baseline physical therapy measures between the groups, slight differences were seen. Specifically, the FB group had a lower mean distance for the 6-minute walk task, compared to the NFB group.

Additionally, the FB and NFB groups did not have statistically significant differences in their baseline average pain, nor their baseline acceptable pain. In terms of demographics, average pain, and measures of physical therapy, these groups were identical in terms of statistical significance differences. The only significant difference at baseline, between these two groups, was on the single sub-section of “POQ Mobility”, on the POQ self-reported questionnaire.

While these groups were similar at baseline, they displayed some differences post-treatment. Namely, the FB group showed a greater decrease in pain intensity after treatment, compared to the NFB group, with a difference that is clinically meaningful. While the FB group showed a greater decrease in pain intensity over time, this change in pain intensity did not strongly correlate with any of the measures of activity collected from the Fitbit, such as the average steps walked over the course of the treatment or the average number of days the Fitbit device was worn. To better understand what may be influencing the greater decrease in pain intensity in the FB group, we analyzed an exploratory logistic regression. The first regression model demonstrated that baseline measures did not display a difference that predicts treatment group. The second regression model, which examined changes between the baseline and follow-up measures, displayed a significant difference between the two groups. In this model, the only significant predictor of treatment group was treatment satisfaction scores. These analyses revealed not only that there were no differences in baseline measurements that led to the FB group having a greater decrease in pain, but that there also were no differences at baseline that we measured, that can be used to attribute why certain patients opted for the Fitbit device while others did not. Despite the lack of statistically significant differences, it is important to note that there were slight differences between the groups at baseline, as previously discussed. However, considering these modest differences and the lack of statistical significance, we offer the following possible interpretations.

One plausible explanation for these findings of greater change in pain intensity in the FB compared to NFB group could be driven by subject bias (defined by the APA as the influence that research participants’ knowledge about aspects of the research has on their responses to experimental conditions and manipulations [27]), as it was up to the patients entering IPRP to decide if they wanted a Fitbit or not. Among those who opted for the Fitbit, their average daily steps and level of engagement with the Fitbit device correlated strongly with some baseline measures (POQ mobility and POQ vitality scores), potentially suggesting that their subjective sense of mobility, energy, and activity levels influenced their decision to opt for the Fitbit device. While this current study did not measure treatment expectation or factors related to emotional affect, such as optimism, hope, or goal-directed activity, we recognize that these may have been elements that led to opting into the Fitbit usage, as well as improved average pain intensity after using the Fitbit across the pain treatment program [28]. Findings from the current observational study could be interpreted in the following ways. First, the patients opting for the Fitbit have hope. They are accepting the Fitbit because they plan to and hope to engage. This is evidenced by the lack of correlation between average daily steps and outcome measures, as well as the lack of correlation between their level of engagement and outcome measures, leaving patient bias as an influencing factor. If this is true, it would mean that those opting for the Fitbit hope they will improve, so in electing for the Fitbit, they feel a greater sense of treatment and more invested in the treatment, resulting in improvements over the course of the treatment, as well as a higher satisfaction in treatment. However, hope was never measured, so the Fitbit device could be serving as a placebo instead. As studied by Kisaalita and colleagues, placebos are most acceptable to patients when the placebo was used as a treatment enhancer or adjunct [29], similar to how the Fitbit was offered as an add-on to the IPRP treatment in this current study. Additionally, the lack of correlation of between average daily steps and level of engagement, compared to treatment outcomes, could be due to detection power or a measurement error. The second interpretation could be that the patients opting for the Fitbit are more engaged. This is evidenced by the patient bias, as it involved engagement on their part to opt in. Unfortunately, we did not have access to pre-treatment activity levels, so while it could be that by seeing and tracking their exercise, they expected better outcomes, we would need data regarding their Fitbit-motivated change in activity to make this determination. Lastly, the third interpretation we can make is that the patients opting for the Fitbit are getting better because they are exercising more. Similar to the second interpretation, this is limited by the lack of pre-treatment activity data, yet no differences in PT measures were noted at baseline, suggesting similar physical abilities.

## Conclusion

We believe this study provides useful clinical information– namely, that there is potential benefit in pain intensity reduction through the implementation of an adjunct wearable activity tracking device in pain treatment programs. However, further studies need to be done in order to better understand why certain individuals opt into wanting the wearable device. Furthermore, additional studies implementing wearable activity trackers should utilize the concept of having activity goals, as studied by Grunberg and colleagues [30].

### Limitations

Physical activity in the non-Fitbit group was not measured, and thus the effects of physical activity can’t be completely ruled out. Physical activity-based treatments for pain have been shown to improve coping with chronic pain, but the physical activity levels of NFB group are unknown.

### Electronic supplementary material

Below is the link to the electronic supplementary material.

Supplementary Material 1

## Data Availability

The datasets used and analyzed during the current study are available from the corresponding author on reasonable request.
